# Paeoniflorin Improved Constipation in the Loperamide-Induced Rat Model via TGR5/TRPA1 Signaling-Mediated 5-Hydroxytryptamine Secretion

**DOI:** 10.1155/2021/6076293

**Published:** 2021-12-08

**Authors:** Yu Zhan, Yong Wen, Liang-liang Zhang, Xu-long Shen, Xiao-hui Chen, Xiao-hai Wu, Xue-gui Tang

**Affiliations:** ^1^Department of Anorectal, Affiliated Hospital of Integrative Chinese Medicine and Western Medicine of Chengdu University of Traditional Chinese Medicine, Chengdu 610042, China; ^2^Department of Anorectal, Chengdu First People's Hospital, Chengdu 610031, China; ^3^Department of Anorectal, Affiliated Hospital of Chengdu University of Traditional Chinese Medicine, Chengdu 610072, China; ^4^Department of Traditional Chinese Medicine, The Affiliated Hospital of Southwest Medical University, Luzhou 646000, China; ^5^Department of Anorectal, Luzhou People's Hospital, Luzhou 646000, China; ^6^Department of Anorectal, Chengdu Shuangliu Hospital of Traditional Chinese Medicine, Chengdu 610200, China; ^7^Department of Integrated Traditional and Western Medicine Anorectal, Affiliated Hospital of North Sichuan Medical College, Nanchong 637000, China; ^8^Chengdu University of Traditional Chinese Medicine, Chengdu 610072, China

## Abstract

Slow transit constipation (STC) is a common type of constipation with a high incidence rate and a large number of patients. We aimed to investigate the therapeutic effects and potential mechanism of paeoniflorin (PAE) on loperamide-induced Sprague Dawley (SD) rat constipation models. Rats with loperamide-induced constipation were orally administered different concentrations of PAE (10, 20, or 40 mg/kg). In vitro, enterochromaffin (EC)-like RIN-14B cells were treated with 20, 40, or 80 *μ*g/ml PAE. We found that PAE treatment significantly improved the symptoms of constipation and increased the intestinal transit rate. Hematoxylin and eosin (H&E) staining showed that PAE alleviated colonic tissue pathological damage. Besides, our results implied that PAE concentration-dependently promoted the content of 5-hydroxytryptamine (5-HT) catalyzed by tryptophan hydroxylase (Tph)-1 in the serum of loperamide-induced rats and in RIN-14B cells. Western blot and immunofluorescence (IF) stain indicated that PAE also promoted the expression of G protein-coupled BA receptor 1 (TGR5), transient receptor potential ankyrin 1 (TRPA1), phospholipase C (PLC)-*γ*1, and phosphatidylinositol 4,5-bisphosphate (PIP2) *in vivo* and *in vitro*. RIN-14B cells were cotreated with a TGR5 inhibitor (SBI-115) to explore the mechanism of PAE in regulating the 5-HT secretion. We observed inhibition of TGR5 reversed the increase of 5-HT secretion induced by PAE in RIN-14B cells. We provided evidence that PAE could promote 5-HT release from EC cells and improve constipation by activating the TRPA1 channel and PLC-*γ*1/PIP2 signaling. Thus, PAE may provide therapeutic effects for patients with STC.

## 1. Introduction

Chronic constipation is a common gastrointestinal complaint characterized by decreased bowel movements or defecation straining, which is considered to affect the quality of life of patients [1]. It affects 2%–27% of the general population in Western countries [2] and 4%–6% in China [3] with a higher prevalence in the elderly and women. In the USA, chronic constipation imposes a substantial economic burden with 1.7 billion in direct and indirect costs [4]. Slow transit constipation (STC) is a common type of chronic constipation caused by abnormalities of colonic motility [5]. STC is mainly manifested as weakened and/or disordered colonic peristalsis and the slow speed of stool pushing forward and emptying. Clinically, purgative abuse is often encountered many times and is especially worsened in the occurrence of STC.

Recently, seeking and developing high-effect, low-poison, and cheap natural medicine for the treatment of constipation has become an international research hotspot. More than two thousand years ago, traditional Chinese medicine (TCM) understood the symptoms of STC and accumulated many documents and experiences in clinical practice over the past dynasties. Thus, TCM has the potential to improve treatment of patients with STC. Paeoniflorin (PAE) is a biologically active ingredient extracted from the dried root of *Paeonia veitchii* Lynch. It was reported that PAE has the effects of antiatherosclerosis, antitumor, treatment of dementia diseases, and immune regulation [6, 7]. Particularly, PAE has been considered as an effective drug for improving digestive system function and treating gastrointestinal injury. Previous studies indicated that PAE prevented intestinal ischemia/reperfusion injury and intestinal barrier disruption via activating LKB1/AMPK signaling-mediated autophagy and anti-inflammation, respectively [8, 9]. However, the function of PAE on STC has not yet been fully investigated.

Many studies found that sensory signals in the intestinal mucosa played an important role in regulating intestinal motility, and their abnormality was closely related with STC progression [10, 11]. The report demonstrated that 5-hydroxytryptamine (5-HT), known as an intestinal neurotransmitter, was abnormally distributed or expressed in the colonic tissues of the STC mice model [12]. Enterochromaffin (EC) cells, which function as chemosensors in the intestinal epithelium, are known to secrete 5-HT, contributing to physiological reactions such as the intestinal peristalsis reflex [13]. Hence, this study investigated the effect of PAE on the improvement of constipation *in vivo* using the loperamide-induced rat model. Furthermore, we also explored the underlying mechanism of PAE regulating the release of 5-HT from EC cells.

## 2. Methods and Materials

### 2.1. Animals and Treatment

30 Sprague Dawley (SD) male rats (SPF grade, 6 weeks) were purchased from Chengdu Dossy Experimental Animals Co., Ltd. (Chengdu, Sichuan) and raised at Chengdu University of TCM. The feeding environment was 25 ± 1°C, relative humidity 50%–60%, and light/darkness for 12 h circulation. SD rats are allowed to eat and drink freely. All experiments were approved according to the Ethics Committee of Chengdu University of TCM.

PAE (purity, >95%) was purchased from Guizhou Dida Technology Co., Ltd. (Guizhou, China). Five different groups of rats were categorized (*n* = 6), namely, control group, model group, 10 mg/kg PAE (PAE low) group, 20 mg/kg PAE (PAE medium) group, and 40 mg/kg PAE (PAE high) group. Constipation was induced by subcutaneous injection of loperamide (3 mg/kg) twice a day for 6 days, and the rats in the control group were injected with saline. After establishing the constipation models, the PAE low group, PAE medium group, and PAE high group were orally administered with 10 mg/kg/d, 20 mg/kg/d, and 40 mg/kg/d PAE, respectively. After 2 weeks of different treatments, all rats were euthanized after being sedated with 1% sodium pentobarbital (50 mg/kg). The colons were extracted and stored at −80°C for further examination.

### 2.2. Measurement of Faecal Output and Water Content

Rat stool pellets were collected for 24 hours and counted, and then the characteristics were assessed. Manure pellets were dried in an incubator at 60°C. The fecal water content was calculated as follows: water content (%) = ((wet weight − dry weight)/wet weight) × 100.

### 2.3. Intestinal Transit Ratio

The intestinal transit ratio was determined as described previously [14]. In brief, after 30 minutes of gavage with PAE on the last day of the experiment, 2 mL of 10% charcoal aqueous suspension was administered orally. And 25 minutes later, the rats were euthanized by cervical dislocation, the small intestines were collected, and the total length and the travel distance of the charcoal in the intestine were measured. The intestinal transit ratio was calculated as follows: intestinal transit ratio = (travel distance of the charcoal in the intestine/the length of the small intestine) × 100.

### 2.4. Hematoxylin and Eosin (H&E) Stain

The colonic tissues of rats were fixed in 4% paraformaldehyde for 24 hours before being embedded in paraffin. The colon was used for histopathological examinations by staining with hematoxylin and eosin (H&E). Then, pathological changes of colonic tissues were examined using the BX70 research microscope (Olympus, Tokyo, Japan).

### 2.5. Cell Culture and Treatment

RIN-14B (a rat pancreatic islet cell line) was obtained from the Shanghai Cell Bank (Shanghai, China). RIN-14B cells were maintained in RPMI1640 medium complemented with 10% FBS at 37°C with 5% CO_2_ in a humidified incubator. For cell processing, cells were treated with PAE (Solarbio, Beijing, China) at different concentrations (20, 40, or 80 *μ*g/ml) for 24 hours.

### 2.6. CCK-8 Assay

RIN-14B cells (2 × 10^4^ cells/well) were seeded into 96-well plates and cultured for 24 hours. Then, the cells were incubated with PAE (20, 40, or 80 *μ*g/ml) for 24 hours. The cell vitality of RIN-14B cells was determined by CCK-8 (Thermo Fisher Scientific) as directed by the manufacturer. Absorbance was recorded at 450 nm.

### 2.7. 5-HT Release Assay

Small pieces of colonic tissue were washed with PBS followed by homogenizing with PBS (10 mg tissue per 100 *μ*L PBS). The resulting suspension was subjected to ultrasonication to further break down the cell membranes, and then homogenates were centrifuged for 15 minutes at 1500 × *g*. The supernatants were collected and stored at −80°C until the 5-HT measurement.

RIN-14B cells were seeded into 6-well plates and cultured for 24 hours. Subsequently, cells were treated with PAE (20, 40, or 80 *μ*g/ml) for 24 hours. The cells were washed with Hank's balanced salt solution (HBSS) containing 0.1% BSA and preincubated with 2 *μ*M fluoxetine (5-HT reuptake inhibitor) for 1 hour. Then, the cells were incubated with 2 ml HBSS containing stimulants for 30 minutes at 37°C. The assay buffer was collected and centrifuged for 3 minutes at 1000 × *g* to remove any detached cells.

The 5-HT concentration in colonic tissues and in the culture supernatant of RIN-14B cells was measured using an ELISA kit (Takara, Japan) as per the manufacturer's instructions. The absorbance was measured at a 450 nm wavelength using an enzyme-linked immune monitor (Thermo Fisher Scientific, Inc., USA).

### 2.8. Immunofluorescence (IF) of Tryptophan Hydroxylase (Tph)-1 and G Protein-Coupled BA Receptor 1 (TGR5)

Paraffin sections of colonic tissues were dewaxed and hydrated. The sections were incubated in QuickBlock™ Blocking Buffer (Beyotime Biotechnology, Jiangsu, China, P0260) for 30 minutes at room temperature. RIN-14B cells were fixed with 4% paraformaldehyde for 30 minutes at 4°C. After cells were washed with PBS three times, they were incubated with 0.5% Triton X-100 for 15 minutes. Then, the sections of RIN-14B cells were incubated with Tph-1 antibody (Abcam, ab228588; 1/150) or TGR5 antibody (Abcam, ab114081; 1/200) at 4°C overnight and washed 3 times with phosphate-buffered saline (PBS). Then, DAPI (Abcam, ab104139; 1/2000) was added dropwise into the sections for 5 minutes. The staining was observed under a fluorescence microscope (BX53 Olympus, Tokyo, Japan) at 100× magnification.

### 2.9. Western Blot Analysis

Colonic tissues or RIN-14B cells were fabricated using RIPA buffer (Signaling Technology, Inc.). The protein concentration was examined by a BCA kit (Sigma-Aldrich; Merck KGaA). Total protein (30 *μ*g/sample) was separated via 10% SDS-PAGE and to nitrocellulose membranes. 5% nonfat dried milk was used to block the membranes. The corresponding protein antibodies were as follows: TGR5 (Abcam, ab72608; 1/1000), transient receptor potential ankyrin 1 (TRPA1; Alomone Labs, ACC-037; 1/1000), phospholipase C (PLC)-*γ*1 (Abcam, ab76155; 1/5000), phosphatidylinositol 4,5-bisphosphate (PIP2; Santa Cruz, sc-53412; 1/200), Tph-1 (Abcam, ab228588; 1/1000), and *β*-actin (Boster, BM0627; 1/1000). Then, the membrane washing was performed with Tris-buffered saline/0.1% Tween (TBST) and incubated for 1.5 hours with an HRP goat antirabbit IgG (Abcam, ab6721). The band visualization was carried out using the ECL system (Affinity Biosciences, Cincinnati, Ohio, USA), and as an internal control, *β*-actin was used.

### 2.10. Statistical Analysis

Means and standard deviations were used to represent the data. SPSS 20.0 (IBM Corp.) was used for statistical analysis. The comparison between groups was done using a one-way analysis of variance (ANOVA) with Tukey's post hoc test of means. *P* < 0.05 were determined as statistically significant.

## 3. Results

### 3.1. Paeoniflorin Concentration-Dependently Accelerated Colonic Motility and Defecation

**Figure 1:**
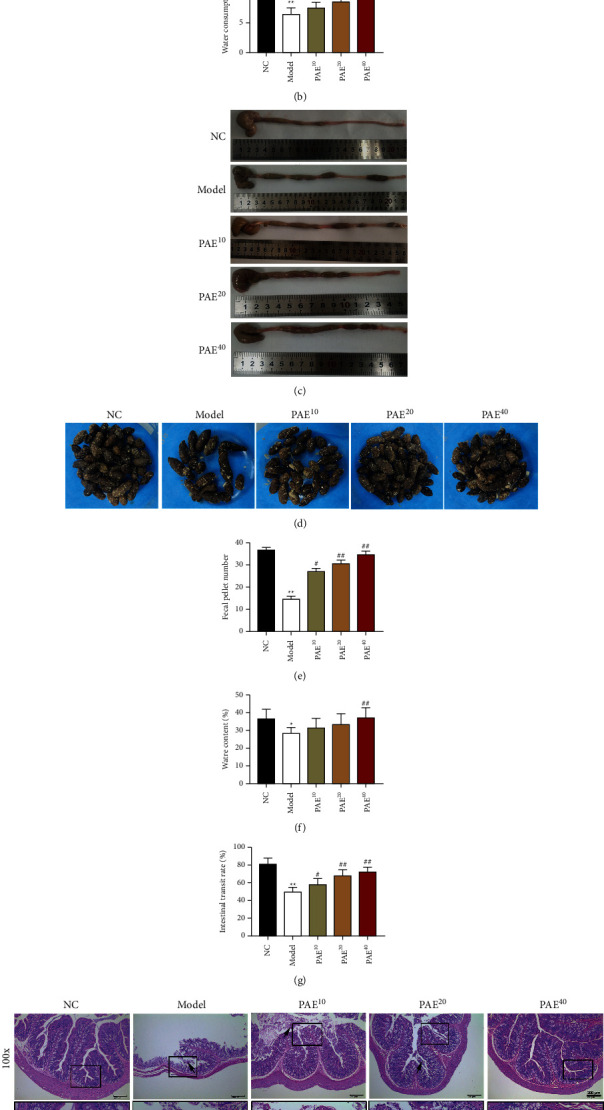
Paeoniflorin concentration-dependently improved constipation and colonic tissue pathology in rats. Food intake (a) and water consumption (b) were tested immediately using an electric balance and measuring cylinder. (c) Visual observation of stool morphology. (d, e) Fecal pellet number was recorded in 24 hours. (f) Water content was measured after drying stools in a 60°C oven for 12 hours. (g) Intestinal transit rate was detected. (h) H&E-stained colonic tissue after administration of PAE (10, 20, and 40 mg/kg) was observed at 100× and 400×. Arrows, mucosal epithelial cell abscission. Data are mean ± SEM. ^*∗*^*P* < 0.05 and ^*∗∗*^*P* < 0.01 vs. NC group; ^#^*P* < 0.05 and ^##^*P* < 0.01 vs. model group.

As shown in Figure 1(a), food intake did not differ significantly among all groups. 20 mg/kg PAE and 40 mg/kg PAE treated rats demonstrated more water consumption than in the normal control rats (Figure 1(b)). Similarly, macroscopic evidence of watery stool in the colon was dramatically observed in PAE (10, 20, and 40 mg/kg)-treated rats compared with the model group (Figure 1(c)). In addition, loperamide-induced rats showed a decrease of fecal pellet number and their moisture content, while PAE treatment concentration-dependently improved these differences (Figures 1(d)–1(f)). Compared with the model group, PAE significantly increased the rate of intestinal transit in a dose-dependent manner (Figure 1(g)). Taken together, these results indicated that administration of PAE improved colonic motility and defecation in constipated rats.

### 3.2. Paeoniflorin Improved the Pathological Changes of Colonic Tissue in Constipated Rats

We then investigated the effect of PAE on structural alteration of the colonic tissue using H&E staining. As shown in Figure 1(h), in the model group, the structure of colonic tissue was damaged, including mucosal epithelial cell necrosis and abscission, inflammatory cell infiltrates in the laminae propria. Fortunately, the structure of the colonic tissue of the rats in PAE-treated groups was gradually recovered to the same level of as that of the control group, and the infiltration of inflammatory cells in the colonic tissue was decreased (Figure 1(h)).

### 3.3. Paeoniflorin Triggered 5-HT Secretion and Tph-1 Expression for Gut Motility

**Figure 2:**
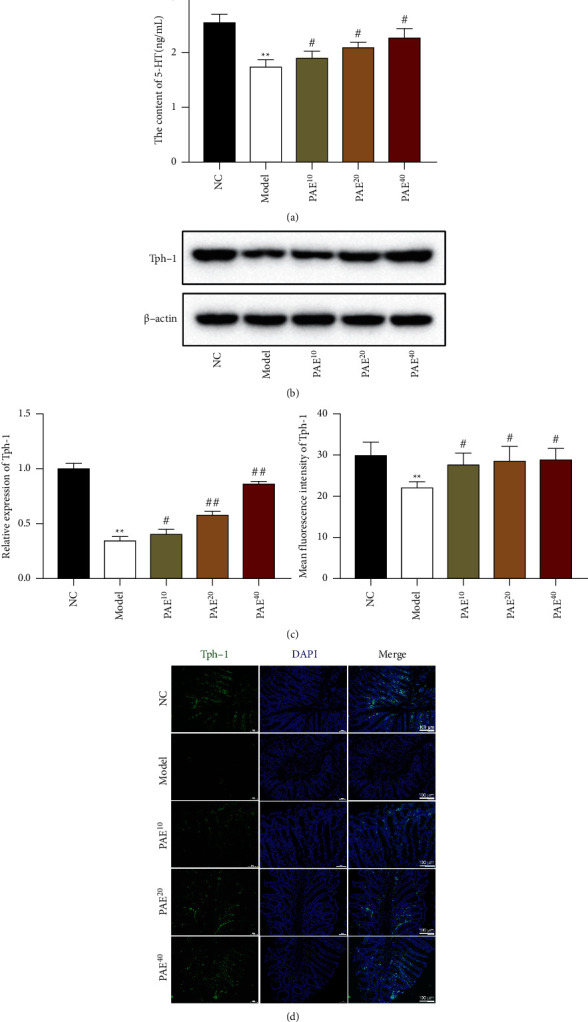
Paeoniflorin triggered 5-HT secretion and Tph-1 expression for gut motility. (a) The 5-HT secretion in the serum of rats was assessed using ELISA. (b–d) The expression of Tph-1 in colonic tissues of rats was tested using western blot and immunofluorescence (IF) stain. Scale 100 *μ*m. Data are mean ± SEM. ^*∗∗*^*P* < 0.01 vs. NC group, ^#^*P* < 0.05 and ^##^*P* < 0.01 vs. model group.

As 5-HT is an important signaling molecule in the gut [15], we investigated the release of 5-HT in the serum of constipated rats. Marked upregulation of 5-HT was observed in the serum of constipated rats, which was reversed by PAE treatment in a dose-dependent manner (Figure 2(a)). Tph-1 expression was reported to reduce in the bowels of patients with gastrointestinal dysfunction, including constipation [16]. And Tph-1 catalyzed the biosynthesis of 5-HT in EC cells [15]. Thus, we next used a western blot assay and IF staining to explore the effect of PAE on Tph-1 expression. As shown in Figures 2(b)–2(d), the expression of Tph-1 was decreased by prucalopride treatment. Moreover, PAE (10, 20, and 40 mg/kg) treatment concentration-dependently hindered a significant decrease in the expression of Tph-1 in the colonic lamina propria of constipated rats (Figures 2(b)–2(d)).

### 3.4. Paeoniflorin Activated TGR5/TRPA1 Signaling Pathway for Gut Motility

**Figure 3:**
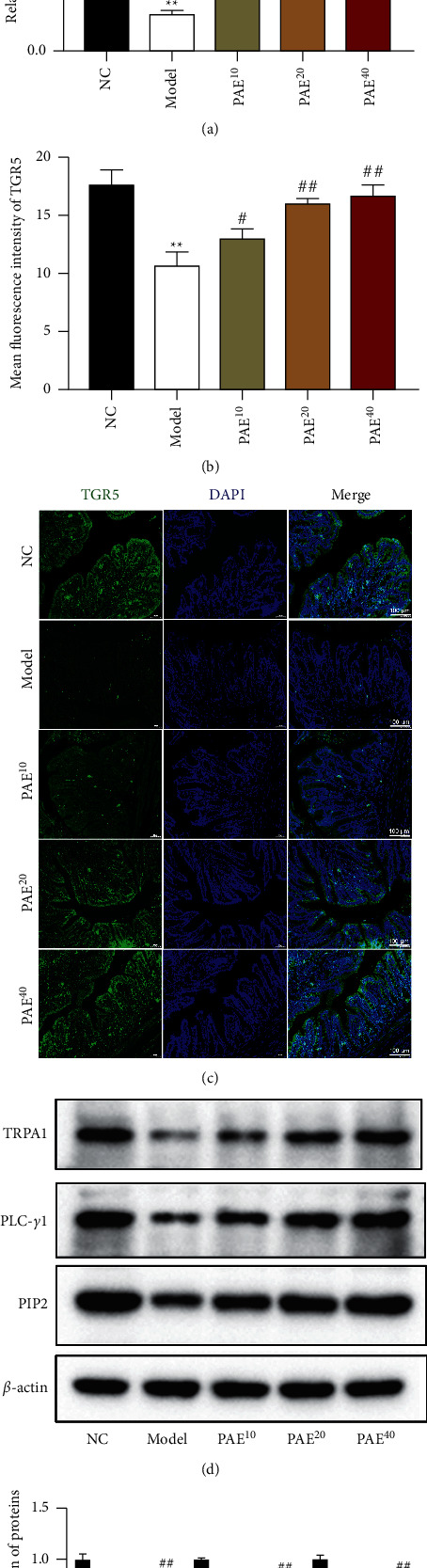
Paeoniflorin activated the TGR5/TRPA1 signaling pathway for gut motility. The protein level of TGR5 in the colonic tissues of constipated rats was determined by western blot analysis (a) and IF stain (b, c). Scale 100 *μ*m. (b) Western blot analysis was used to examine the protein expression of TRPA1, PLC-*γ*1, and PIP2 in colonic tissues of rats. Data are mean ± SEM. ^*∗∗*^*P* < 0.01 vs. NC group; ^#^*P* < 0.05 and ^##^*P* < 0.01 vs. model group.

TGR5, as a metabolic regulator, was involved in energy homeostasis and control of gastrointestinal motility [17]. We further found that the expression of TGR5 was significantly decreased in the loperamide-induced group than that in the control group (Figures 3(a)–3(c)). However, PAE treatment markedly concentration-dependently increased TGR5 expression in the mucosal epithelium of colonic tissues (Figure 3(a)–3(c)). Indeed, the TRPA1 channel, a downstream regulatory protein of TGR5, was reported to induce 5-HT release in EC cells [17]. Western blot assay showed that the protein expression of TRPA1, PLC-*γ*1, and PIP2 were attenuated in colonic tissues of constipated rats (Figures 3(d) and 3(e)). Consistently, we observed enhanced TRPA1, PLC-*γ*1, and PIP2 expression in PAE-treated groups (Figures 3(d) and 3(e)).

### 3.5. Paeoniflorin Induced TGR5/TRPA1 Signaling Activation for 5-HT Release and Tph-1 Expression in EC Cells

**Figure 4:**
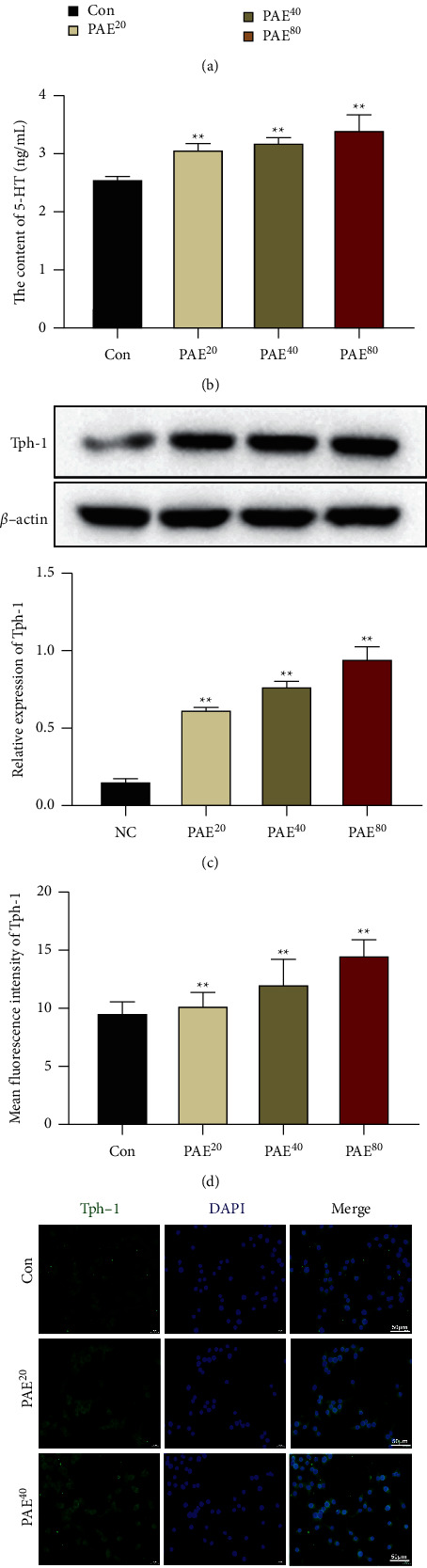
Paeoniflorin induced 5-HT release and Tph-1 expression in EC cells. RIN-14B cells were treated with different concentrations of PAE (20, 40, or 80 *μ*g/ml) for 24 hours. (a) The CCK-8 assay was used to detect cell proliferation of RIN-14B cells at 24 hours and 48 hours. (b) The 5-HT secretion in the cell supernatant of RIN-14B cells was tested by ELISA. The expression of Tph-1 in RIN-14B cells was checked by western blot (c) and IF stain (d, e). Scale 50 *μ*m. Data are mean ± SEM. ^*∗∗*^*P* < 0.01 vs. Con group.

Since EC cells are the main source of peripheral 5-HT, we then investigated the role of PAE in EC cells. Different concentrations of PAE (20, 40, or 80 *μ*g/ml) were used to treat RIN-14B cells, which are considered to be a model for EC cells due to 5-HT secretion. The CCK-8 assay indicated that 20 *μ*g/ml and 40 *μ*g/ml PAE increased cell proliferation compared with the control group at the first 24 hours, and at 48 hours, cell proliferation was dose-dependently promoted by PAE treatment (Figure 4(a)). Meanwhile, 5-HT secretion was increased in PAE treated-RIN-14B cells in a dose-dependent manner (Figure 4(b)).

**Figure 5:**
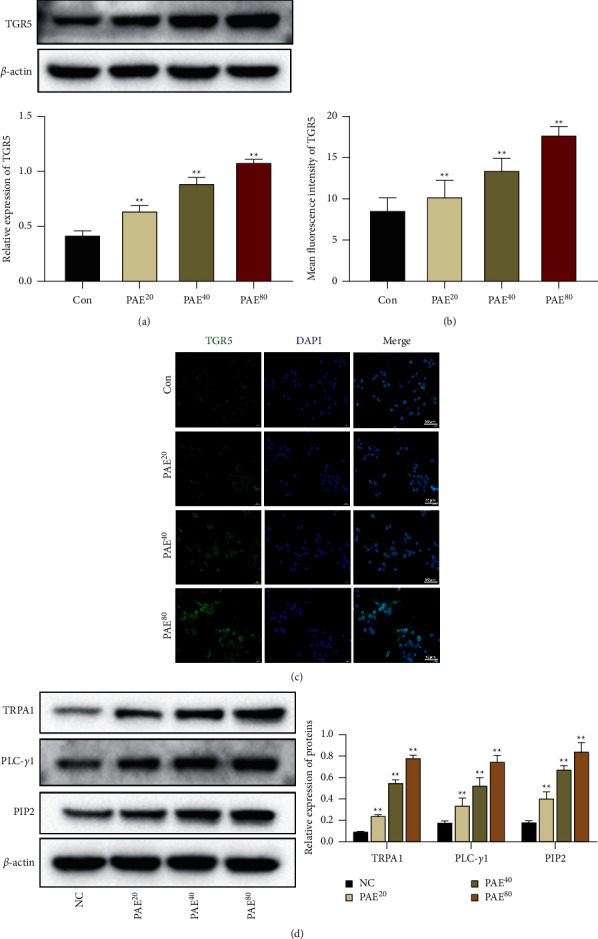
Paeoniflorin promoted TGR5/TRPA1 signaling activation in EC cells. RIN-14B cells were treated with different concentrations of PAE (20, 40, or 80 *μ*g/ml) for 24 hours. (a) The expression of TGR5 was detected by western blot. (b, c) The expression of TGR5 was tested by IF stain. Scale 50 *μ*m. (d) The expression of TRPA1, PLC-*γ*1, and PIP2 in RIN-14B cells was detected by western blot. Data are mean ± SEM. ^*∗∗*^*P* < 0.01 vs. Con group.

Then, we used western blot and IF analysis to check for changes in Tph-1 expression and TGR5/TRPA1 signaling activation. As shown in Figures 4(c)–4(e), PAE treatment dose-dependently activated Tph-1 expression in RIN-14B cells compared with the control group. Additionally, the protein levels of TGR5, TRPA1, PLC-*γ*1, and PIP2 were also increased by PAE treatment (Figures 5(a)–5(d)). Therefore, we hypothesized the TGR5/TRPA1 signaling pathway mediated the regulatory effects of PAE on 5-HT release from EC cells.

### 3.6. TGR5/TRPA1 Signaling Activation Was Required for PAE-Induced 5-HT Release and Tph-1 Expression

**Figure 6:**
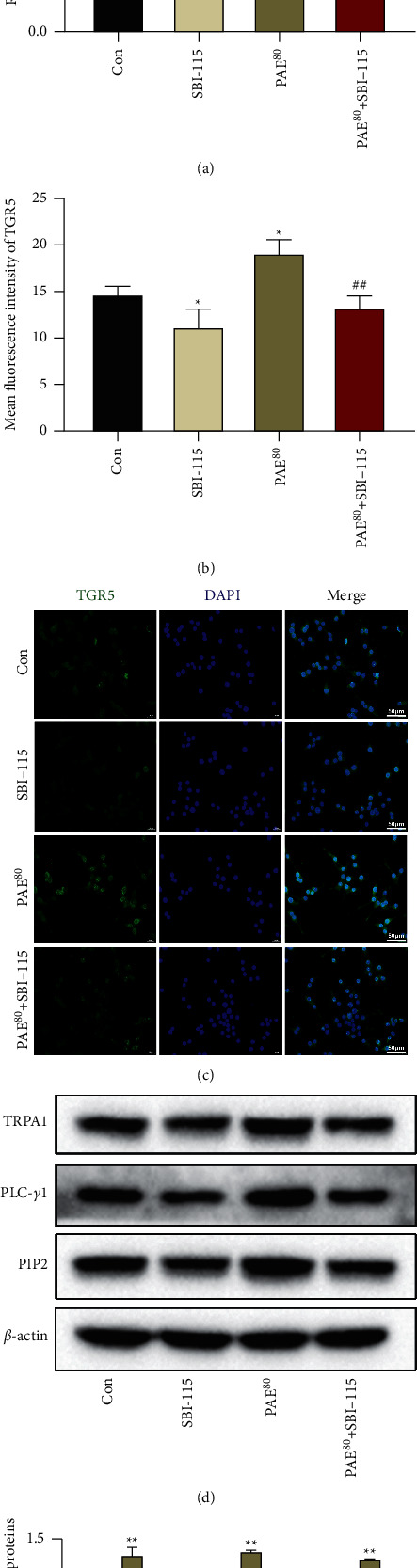
TGR5/TRPA1 signaling activation was inhibited by SBI-115. The protein level of TGR5 in RIN-14B cells was detected by western blot analysis (a) and IF stain (b, c). Scale 50 *μ*m. (d, e) Western blot analysis was used to determine the protein levels of TRPA1, PLC-*γ*1, and PIP2 in RIN-14B cells. Data are mean ± SEM. ^*∗*^*P* < 0.05 and ^*∗∗*^*P* < 0.01 vs. Con group; ^##^*P* < 0.01 vs. PAE^80^ group.

**Figure 7:**
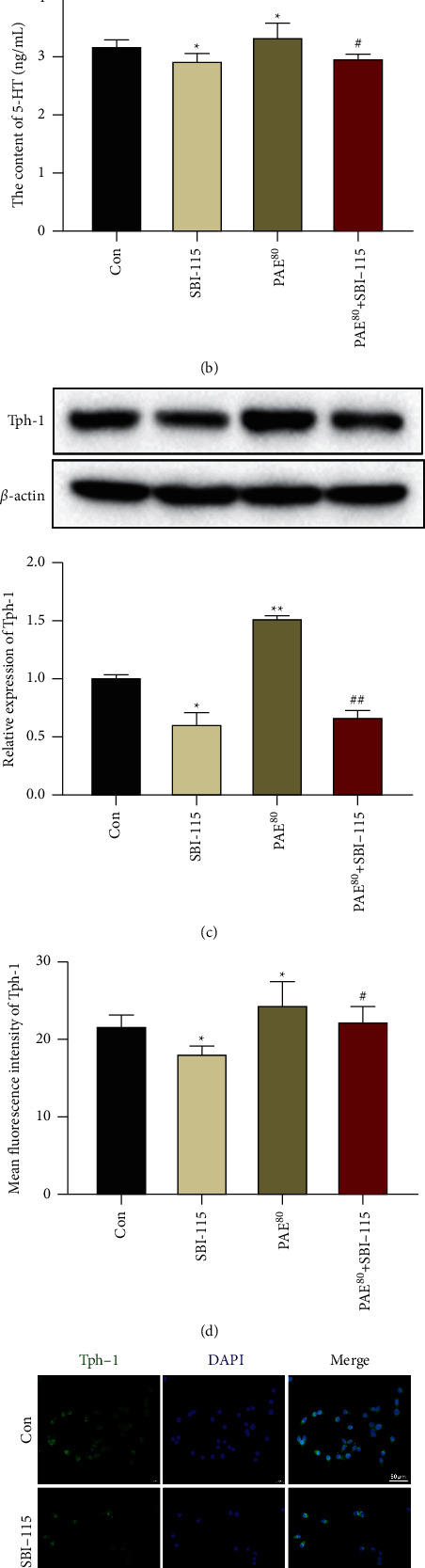
5-HT release and Tph-1 expression were hindered by TGR5/TRPA1 signaling deactivation. (a) The CCK-8 assay was used to test the cell proliferation of RIN-14B cells at 24 hours. (b) The 5-HT secretion in the cell supernatant of RIN-14B cells was assayed by ELISA. The expression of Tph-1 in RIN-14B cells was determined via western blot (c) and IF stain (d, e). Scale 50 *μ*m. Data are mean ± SEM. ^*∗*^*P* < 0.05 and ^*∗∗*^*P* < 0.01 vs. Con group; ^#^*P* < 0.05 and ^##^*P* < 0.01 vs. PAE^80^ group.

Finally, a TGR5 inhibitor SBI-115 was cotreated with PAE (80 *μ*g/ml) to verify the mechanism of PAE on 5-HT release and Tph-1 expression in RIN-14B cells. We found that the increases of TGR5, TRPA1, PLC-*γ*1, and PIP2 expression in PAE treated-RIN-14B cells were significantly weakened by SBI-115 (Figures 6(a)–6(e)). Meanwhile, SBI-115 silted the increase of RIN-14B cell proliferation induced by PAE treatment (Figure 7(a)). As shown in Figures 7(b)–7(e), the 5-HT release and Tph-1 expression were significantly decreased in SBI-115 cotreated-RIN-14B cells compared with that in PAE treated-RIN-14B cells. These data suggest that PAE promoted 5-HT release and Tph-1 expression through stimulating the TGR5/TRPA1 signaling pathway in EC cells.

## 4. Discussion

Loperamide is a common antidiarrheal drug in the clinic. The studies suggested that loperamide could inhibit intestinal motility and intestinal fluid secretion through several mechanisms such as blocking acetylcholine release [18] and calcium channels [19], as well as inhibiting the function of calmodulin [20]. Thus, loperamide is extensively used to establish animal constipation models [21–23]. In the present study, we successfully established a constipation rat model using loperamide treatment, as evidenced by decreases in fecal pellets, fecal water content, and intestinal transit rate. And we observed that PAE could improve these symptoms of constipated rats and relieve the pathological changes of colonic tissues in a concentration-dependent manner. We confirmed that 40 mg/kg was the best dose of PAE in our experiment.

Enterochromaffin cells are located throughout the mucosal layer of the gastrointestinal tract [24]. As mechanical and chemical sensors, they can synthesize and release peripheral 5-HT and ultimately participate in many pathophysiological processes of the gastrointestinal tract [25, 26]. 5-HT is an important neurotransmitter, which binds to the corresponding receptors in the enteric nervous system (ENS) and smooth muscle epithelial cells and ultimately participates in the regulation of gastrointestinal motility and secretion functions [27, 28]. Strikingly, recent studies in the literature have reported the different regulatory effects of PAE on 5-HT secretion. PAE significantly attenuated chronic unpredictable stress (CUS)-induced reductions of 5-HT and its metabolite 5-hydroxyindoleacetic acid (5-HIAA) in rats' serum [29]. Meanwhile, PAE also significantly enhanced the expression of 5-HT and 5-HIAA in the hippocampus of Institute of Cancer Research (ICR) mice, which implied that PAE had antidepressant-like effects [30]. A study showed that PAE reduced the content of 5-HT in the medulla of a mouse brain, whereas it expanded the content of 5-HIAA in anesthetic rat cortex and striatum [31]. In addition, PAE regulated hepatocellular carcinoma (HCC) development through inhibiting the level of 5-HT receptor 1D (5-HT1D) in HepG2 and SMMC-7721 hepatoma cells [32]. Taken together, PAE had the opposite effect on the regulation of 5-HT secretion and its receptor expression. The reason may be that PAE simultaneously regulates the expression of multiple enzymes that mediate the synthesis of 5-HT. Tryptophan hydroxylase (Tph) is recognized to be a rate-limiting enzyme for 5-HT synthesis, which catalyzes the production of 5-HT from the essential amino acid tryptophan [33]. Tph includes two types Tph-1 and Tph-2 to involve in the synthesis of 5-HT. Intriguingly, Tph-1 is mainly expressed by EC cells and by other non-neuronal cell types such as adipocytes, which affects the synthesis of peripheral 5-HT [34]. Tph-2 is primarily found in the brain stem and mesenteric nerve cells, which affects the synthesis of 5-HT in the central nervous system [35]. In our results, we found that PAE promoted Tph-1 expression and 5-HT secretion in the serum of loperamide-induced rats and in RIN-14B cells. These findings indicate that PAE specifically increased the content of 5-HT catalyzed by Tph-1 in EC cells.

TGR5, a member of the G protein-coupled receptor (GPCR) family, is recognized to be a membrane receptor for bile acids which consists of 330 amino acids forming 7 transmembrane structural domains [36]. TGR5 is ubiquitous in human organs and tissues, including the spleen, lung, liver, kidney, gastrointestinal tract, bone marrow, and so on [37, 38]. As a metabolic regulator, TGR5 plays an important role in bile acid metabolism, glucose metabolism, and energy homeostasis, as well as inflammatory response regulation [17]. Moreover, the function of TGR5 was also extended to intestinal dynamics regulation. And its agonists may be considered as therapeutic options for digestive disorders [39, 40]. TGR5 mediated the prokinetic actions of intestinal bile acids, and its deficiency caused constipation in mice [41]. In TGR5-overexpressed mice, colonic transit time was reduced, and the defecation frequency was increased [41]. In the intestine, TGR5 was expressed in 5-HT storing EC cells and enteric neurons that firmly implicated it in the release of 5-HT [42]. Our data indicated that PAE increased TGR5 expression in the colonic tissue of constipated rats and in EC cells. And inhibition of TGR5 significantly reduced 5-HT secretion from EC cells.

Here, we also observed that PAE promoted the activation of the TRPA1 channel and PLC-*γ*1/PIP2 expression, which were obstructed by the TGR5 inhibitor. TRPA1 is a sensor molecule on the EC cell membrane, which can sense mechanical and chemical stimuli in the intestinal cavity, mediate the release of 5-HT from EC cells, and thereby regulate gastrointestinal motility [13]. GPCRs (including TGR5) have been shown to modulate the activation of phospholipase C (PLC) -*γ*1/PIP2/TRPA1 signaling cascade [43]. And TGR5 overexpression activated the TRPA1 channel to exacerbate itch in mice [44]. Meanwhile, the study found that the IL-33-ST2 axis stimulated PLC-*γ*1/TRPA1 signaling in EC cells for 5-HT release, thereby maintaining intestinal homeostasis [45]. Our research provided evidence that PAE could promote 5-HT release from EC cells and improve constipation by activating the TRPA1 channel and PLC-*γ*1/PIP2 signaling.

## 5. Conclusions

In conclusion, the present findings identify PAE as significantly improving the symptoms of constipation in rats and promoting the release of 5-HT from EC cells. And its molecular mechanism was to promote the activity of the TGR5/TRPA1 signaling pathway. Our results provided the rationale for preclinical studies of PAE as a potential therapy for STC.

## Data Availability

The datasets used or analyzed during the current study are available from the corresponding author on reasonable request.
